# Research on Multi-Alternatives Problem of Finite Element Model Updating Based on IAFSA and Kriging Model

**DOI:** 10.3390/s20154274

**Published:** 2020-07-31

**Authors:** Juntao Kang, Xueqiang Zhang, Hongyou Cao, Shiqiang Qin

**Affiliations:** School of Civil Engineering and Architecture, Wuhan University of Technology, Wuhan 430070, China; jtkang@163.com (J.K.); caohongyou0625@163.com (H.C.); shiqiangqin@whut.edu.cn (S.Q.)

**Keywords:** finite model updating, multi-alternatives problem, improved artificial fish swarm algorithm, Kriging model

## Abstract

Due to insufficient test data, insufficient constraint equations and uncertain objective function, the local optimal solution and the global optimal solution of the objective function in finite element model updating may represent the actual parameters of the structure. Based on this, this paper proposes an improved artificial fish school algorithm. By combining the niche technology with the artificial fish school algorithm, the improved algorithm can systematically find multiple global optimal solutions and local optimal solutions of the objective function. Aiming at the difficulty of determining the niche radius, an adaptive niche radius mechanism is proposed. The improved algorithm is used to study the multi-alternatives problem of finite element model updating after verifying its feasibility through numerical simulation analysis. In the case of benchmark framework model updating, it is confirmed that multi-alternative problems exist and the global optimal solution of the objective function does not necessarily represent the true parameters of the structure. In case 2, the improved algorithm combined with the Kriging model is applied to the model updating of a cable-stayed footbridge, and 15 sets of solutions are obtained, in which the error objective function values of the measured and theoretical values of the bridge modes are close but the solutions are completely different. Combining with the actual bridge condition and reanalysis technology, the author takes the suboptimal solution 2 as the most representative solution of the bridge parameters, which reduces the possibility of misjudgment of structural parameters.

## 1. Introduction

The finite element model is usually established according to the design drawings. In the process of modeling, there are geometric parameters, physical parameters, boundary conditions, and other assumptions, which make the theoretical values of the model have errors with the actual responses of the structure. The accurate finite element model is very important for the evaluation of the seismic and wind resistance performance, structural damage identification, and health condition monitoring of the existing structure [1,2,3]. In order to obtain the benchmark model, the finite element model updating (FEMU) aimed at reducing the structural theoretical responses and the actual responses error has been extensively studied by scholars at home and abroad. The research review and results of FEMU can be found in the literature [4,5,6,7,8,9,10,11,12,13,14]. In summing up the research progress of FEMU, Li et al. [7] reviewed the main methods of traditional FEMU and pointed out that the FEMU is developing from linear to nonlinear. Teughels et al. [12] carried out the FEMU research earlier; he proposed a method named coupled local minimizers, which was applicable to global optimization of functions with multiple local minima. Then, the proposed method was applied to the finite element model updating of a reinforced concrete beam, and the damage status of the beam was identified successfully. Stana et al. [13] found that even a very detailed initial FEM could not guarantee the actual response of the structure, by updating the FEM of a footbridge, the error between the theoretical responses and the measured responses of the structure are reduced. Plenty of research on FEMU has made this technology develop rapidly, although FEMU has been successfully applied to the field of engineering, the rapid development of engineering technology has put forward higher requirements for FEMU. Some research results show that the updated finite element model based on global optimal solution still cannot guarantee the agreement with the actual structural response [15,16,17,18,19,20,21,22]. In fact, under the influence of environmental noise, bearing friction and other factors, both the test data and model parameters are uncertain, so the results of FEMU based on the determined test data, the determined objective function and the determined parameters to be updated are only a special case, there may be other alternatives that represent the real structural parameters better than the global optimal solution [15,16]. Therefore, the traditional FEMU that only takes a set of global optimal solutions as the real parameters of the structure is unreasonable. Modeling to generate alternatives (MGA), which can produce multiple alternatives, is born. The objective function values of other global optimal solutions or local optimal solutions found by MGA are the same or similar to the global optimal solutions, but they are in different positions. The decision-maker can make comprehensive analysis of multi-alternatives according to engineering experience and reanalysis technology, and then make a choice to reduce the possibility of misjudgment of the actual parameters of the structure [17,18].

In recent years, MGA has been extended to the field of civil engineering. Although some progress has been made in the field of FEMU, it still faces the following problems: (1) difficult in finding multiple alternatives of the error objective function and selecting a set of “correct solution” from the multiple solutions [17,18,19,20,21,22]; (2) low computation efficiency [23,24,25,26,27,28,29,30]. Regarding the first question, relevant scholars carried out the following research: Zárate et al. [18] proposed a FEMU technology based on hop skip and jump algorithm (HSJA), which can find multiple solutions of the objective function. Then HSJA was applied to the model updating of a cable-stayed bridge, and good results were obtained; Caicedo et al. [17] proposed a steady-state genetic algorithm (SSGA) which can find the global and local optimal solutions of multi-peak functions. After verifying the performance of the algorithm, it was applied to the FEMU of the American Society of Civil Engineers(ASCE) benchmark model. The results showed that the global optimal solution of the objective function was not the real damage of the structure, on the contrary, the local optimal solution was the most representative of the real parameters of the structure. Neither the HSJA nor the SSGA can find multiple peaks of multi-peak functions systematically. The improved algorithm proposed in previous research [19,20] also has the phenomenon of missing solutions due to the poor setting of the niche radius. The “correct solution” may be missed due to the poor performance of the algorithm in finding multiple solutions; in addition, the existing research of MGA focuses on the model updating of laboratory structures, the decision-makers can easily choose the “right key” from multiple alternatives by preset damage [21,22,23]. However, due to the complexity of the actual engineering structure, few scholars apply MGA to the updating of large-scale structural models. It is even more challenging to choose a set of “right key“from the multiple solutions of objective function. In order to solve the problem of low efficiency, some scholars proposed to use a proxy model instead of the finite element model (FEM) to reduce the time-consuming aspects of model updating. The commonly used proxy models are the response surface model, Kriging model, and neural network proxy model [24,25,26]. The Kriging model is composed of polynomial regression model and random refinement simulation, which can fit high-order nonlinear problems, and has been successfully applied to geological, mechanical engineering, aerospace, and other fields [27,28]. However, the Kriging model is still limited in the field of large-scale structural FEMU. In order to determine the robustness and effectiveness of the Kriging model for complex full-scale bridge structure, further research is still needed [29,30].

The above problems and challenges stimulate the author to further study multi-alternatives problem of FEMU. This paper is summarized as follows: Section 1 introduces the FEMU and multi- alternatives problem, and briefly introduces the relevant theory of the Kriging model used in this paper, and gives the research flow of multi-alternatives problem of FEMU; Section 2 will introduce the improved artificial fish swarm algorithm and verify its feasibility through numerical simulation analysis; Section 3 applies the improved algorithm to the updating of ASCE benchmark model, and demonstrates that the multi- alternatives problem of the FEMU exists. Next, the improved algorithm is combined with the Kriging model to update the model of a full-scale footbridge. Finally, conclusions are drawn.

## 2. Theory

### 2.1. FEMU and Multi-Alternatives Problem

The process of FEMU is to obtain the structural response information Y first, and then deduce the structural parameter X according to the response information [22]. Therefore, FEMU is an inverse problem, which can be expressed mathematically as shown in Equation (1).

(1) $$X=f^{-1}(Y)$$

In order to obtain the real parameters of the actual FEM of the structure, the error between the measured value of the structure and the theoretical value of the FEM is usually reduced to the greatest extent. The expression of the objective function is shown in Equation (2).

(2) $$\min J(x)=\min\sum_{i=1}^{m}\alpha_{i}r_{i}(x)$$

where $r_{i}(x)$ means the residual function that expresses the relative difference between the analytically predicted response and the experimentally measured response, $\alpha_{i}$ represents the weight value of each response. The response can be modal frequency, nodal displacement, nodal acceleration, etc., represented only by reducing the error between the measured modal frequency of the structure and the theoretical frequency of FEM; the expression is shown in Equation (3).

(3) $$\min J(x)=\min\sum_{j=1}^{n}|\frac{f_{m}^{j}(x)-f_{e}^{j}}{f_{e}^{j}}|,\;x\in [VLB,VUB]$$

where *x* is the value of the parameter to be modified, *VLB* and *VUB* are the upper and lower limits of parameter values, respectively, $f_{m}^{j}(x)$ represents the *j*-th frequency calculated by FEM, $f_{e}^{j}$ means the *j*-th frequency measured by the experiment, and *m* is the modal order.

**Figure 1:**
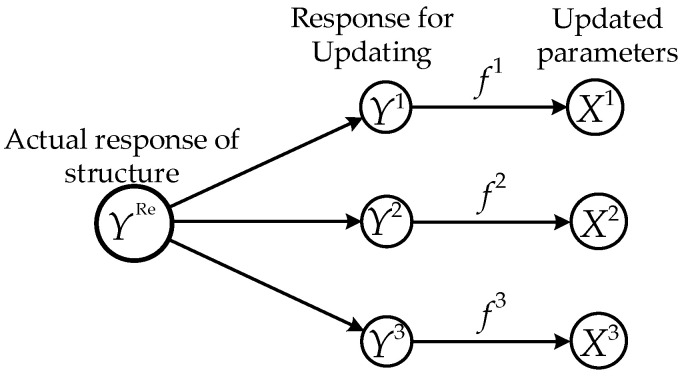
Schematic diagram of multi-alternatives in model updating.

The diversity of the measured response information and the uncertainty of updating parameters determine that the objective function constructed in the FEMU is uncertain, and the optimal parameters obtained by different objective functions may not be the same. The schematic diagram of multi-alternatives caused by different response information is shown in Figure 1.

The following reasons may also lead to the multiple solutions problem of finite element model updating:

(1) In the process of structural test, an insufficient number of sensors leads to incomplete test data, and the objective function in finite element model updating may correspond to multiple solutions due to insufficient constraint equations;
(2) There may be errors in the experimental data. When the objective function is constructed by the experimental data with errors, the global optimal solution may be a set of deceptive solutions. On the contrary, the local optimal solution of the objective function may best represent the real parameters of the structure;
(3) The finite element model updating is an inverse problem in mathematics. Due to the complexity of the finite element model, one response may correspond to multiple solutions.

**Figure 2:**
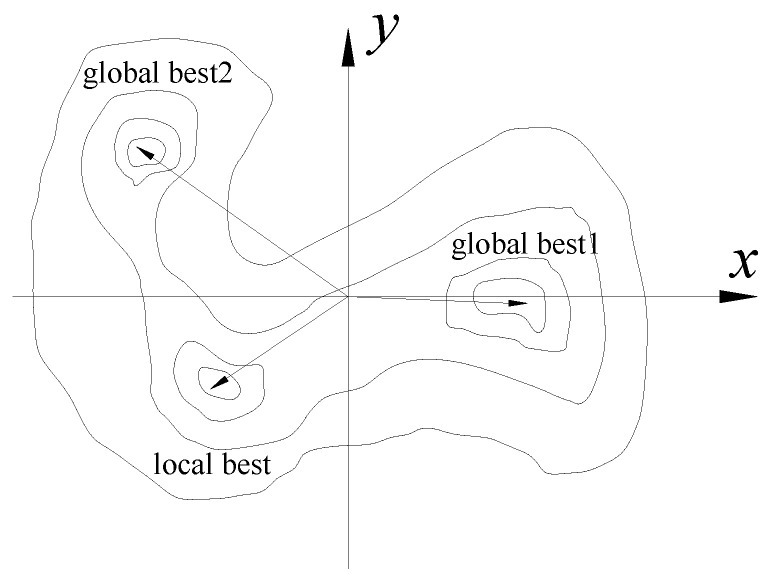
Feasible region of bivariate in model updating.

In fact, even if the objective function and updating parameters are determined, the same objective function value may correspond to multiple solutions due to the complexity of the FEM. Taking the two updating parameters as an example, the objective function *f* simultaneously has two global optimal solutions (*x*_1_, *y*_1_) and (*x*_2_, *y*_2_), and simultaneously satisfies Equation (4), as shown in Figure 2. For a local optimal solution (*x*_3_, *y*_3_) that exists, if Equation (5) is satisfied, it will be used as an alternative. In the formula, *ε* is the threshold value, which controls whether to accept the suboptimal solution.

(4) $$f(x_{1},y_{1})=f(x_{2},y_{2})=\min(f(x,y))$$

(5) $$|f(x_{3},y_{3})-\min(f(x,y))|=\varepsilon$$

Therefore, it is not reasonable for the traditional FEMU technology to take a group of global optimal solutions as the most representative structural actual parameters, so it is urgent to find a multi-alternatives technology in the FEMU. Through this technique, we can find multiple global optimal solutions or local optimal solutions which can reduce the error between the measured value and the theoretical value. The corresponding objective function values of multiple solutions are similar, but the solution space is quite different. The decision-makers can select one or more groups based on the comprehensive analysis of engineering experience, which can not only effectively avoid the misjudgment of structural parameters, but also carry out the risk assessment and reliability calculation of the structure by combining multi-alternatives.

### 2.2. Kriging Model

Kriging model is composed of two parts, one is the deterministic polynomial regression model, which can well fit the linear and low-order nonlinear model problems, the other is the stochastic refinement simulation process using Gaussian function to fit the high-order nonlinear model. The expression of the Kriging model is shown in Equation (6).

(6) $$y(x)=\sum_{i=1}^{p}\beta_{i}f_{i}(x)+z(x)=f^{T}(x)\beta +z(x)$$

where *x* and *y*(*x*) represent input and output responses respectively, *f*^T^(*x*)*β* is polynomial regression model, *β* indicates the coefficient of polynomial vector regression, and *z*(*x*) refines the simulation part randomly with Gaussian function.

To construct the Kriging model, we need to obtain test samples, usually adopting the design methods of central composite design, Box–Behnken design, and Latin hypercube design (LHD) [31]. After the Kriging model is built, its fitting accuracy needs to be tested. Generally, the determination coefficient R^2^, root mean square error (RMSE), mean square error (MSE), and other methods are used to test, and it can only be used after meeting the accuracy requirements.

### 2.3. Procedure of Multi-Alternatives of FEMU

This section summarizes the detailed procedure of Kriging model assisted multi-alternatives FEMU. The whole approach can be divided into the following six steps:

Step 1: An initial FEM using commercial FE software is established based on design documents;

Step 2: Determine the updating parameters by combining engineering experience and parameter sensitivity analysis or parameter significance analysis;

Step 3: Use Latin hypercube design to generate samples, and the samples are substituted into the finite element model to extract the corresponding responses;

Step 4: The Kriging model to describe the relationship between structural responses and updating parameters is established and test the fitting accuracy of the proxy model. If the accuracy of the proxy model meets the requirements, go to the next step, otherwise return to step 3;

Step 5: Construct the error objective function between the responses of the Kriging model and the measured values of the structure;

Step 6: Use the improved artificial fish swarm algorithm proposed in this paper to optimize the parameters and gets the objective function’s multiple solutions;

Step7: Evaluate the results of FEMU and comprehensively analyze the multiple solutions, trying to select one or more solutions that best represent the structural damage parameters.

**Figure 3:**
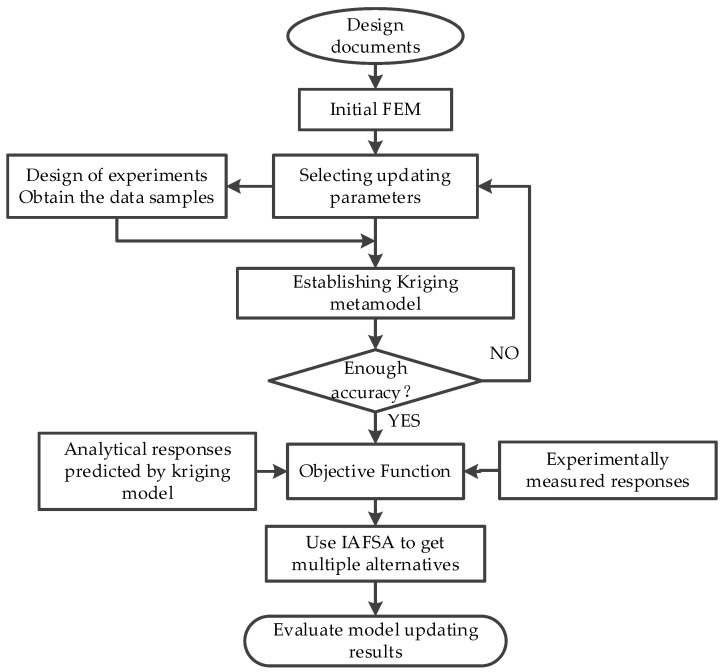
The research flow of multi-alternatives problem in finite element model updating (FEMU).

The flow chart is shown in Figure 3.

## 3. Improved Algorithm and Numerical Simulation Analysis

### 3.1. Standard Artificial Fish Swarm Algorithm

Artificial fish swarm algorithm (AFSA) [32] is one of many swarm intelligence algorithms, which simulates the behavior of a fish swarm always moving in the direction of high food concentration and low crowding degree and has the advantages of strong robustness and global search ability. The principles of AFSA are as follows:

(1) Foraging behavior. Fish search other fish in the field of vision within the maximum number of attempts until they find the fish that is better than the current situation and move in this direction. The mathematical expression is shown in Equation (7):
  (7) $$x_{i}(\mathrm{next})=x_{i}+\mathrm{rand}\times \mathrm{step}\times \frac{x_{j}-x_{i}}{\|x_{j}-x_{i}\|}$$
(2) Swarming behavior. The fish find the central coordinate in the field of vision. If the food concentration is better than the current situation and not crowded, they will swim to the central coordinate. The mathematical expression is shown in Equation (8):
  (8) $$x_{i}(\mathrm{next})=x_{i}+\mathrm{rand}\times \mathrm{step}\times \frac{x_{c}-x_{i}}{\|x_{c}-x_{i}\|}$$
(3) Following behavior. The fish move towards the fish with the highest food concentration in the field of vision. The mathematical expression is shown in Equation (9):
  (9) $$x_{i}(\mathrm{next})=x_{i}+\mathrm{rand}\times \mathrm{step}\times \frac{x_{\mathrm{best}}-x_{i}}{\|x_{\mathrm{best}}-x_{i}\|}$$
(4) Random behavior. When the artificial fish does not satisfy the above three behavioral conditions, it will perform a random swimming behavior, the expression of which is shown in Equation (10):
  (10) $$x_{i}(\mathrm{next})=x_{i}+(2\times \mathrm{rand}-1)\times visual$$
  where *x*_i_ (next) is the next position of the fish, *x_i_* is the current position, *x_j_* is the fish better than the current position, *x*_c_ is the coordinate center of the fish in the field of vision, and *x*_best_ is the optimal coordinate in the field of vision. Step means the moving step, and visual indicates the field of vision.

### 3.2. Improved Artificial Fish Swarm Algorithm

AFSA has been successfully applied in aerospace, civil engineering, communication engineering, and other fields [33,34,35,36]. For example, Li et al. [33] applied AFSA to FEMU and structural damage detection. The results show that the structural damage detection method based on AFSA can effectively modify FEM of the structure and accurately identify the damage degree under different noise levels and damage conditions. Zhang et al. [34] introduced crossover operator and Gaussian mutation operator in genetic algorithm into AFSA, which accelerated the convergence speed of AFSA and improved its optimization accuracy. Then, the improved AFSA was applied to FEMU of an aircraft model, and good results were obtained. Zhu et al. [35] found that the convergence of AFSA will be slow or even stagnant in the process of optimization. Therefore, an improved method using U-shaped function transformation to deal with the random swimming step of artificial fish is proposed, which also improves the optimization accuracy and convergence speed of AFSA. Then, the improved AFSA is applied to the model updating of a cable-stayed bridge, which makes the theoretical responses of the structure match the measured responses.

The above improvement measures can either accelerate the convergence speed or improve the global optimization ability of AFSA. However, the improved algorithm can only find a global optimal solution of multi-peak functions, which is not applicable to the multi alternatives problem of FEMU. Therefore, the standard AFSA still has the following defects: (1) difficult in finding multiple global optimal solutions and suboptimal solutions for multi-peak functions; (2) when AFSA runs to later stage, the artificial fish oscillates near the global optimal solution, and the optimization accuracy is low. In view of the above two problems, the authors propose an improved artificial fish swarm algorithm (IAFSA). The improvement measures are as follows:

(1) In view of the difficulty of finding multiple peaks in the multi-peak function of AFSA, the sequential niche technology is combined with AFSA, and the niche radius *R* is introduced to control the distance between the fish. The specific steps are as follows:

Step 1: Execute AFSA once, find a global optimal solution, and liberate it into the niche core;

Step 2: Repeat AFSA, calculate the distance between the current fish and the existing solution in the niche core, and compare the distance with the radius of each niche, if within the radius, initialize the artificial fish position;

Step 3: Calculate the distance between the optimal solution obtained by each AFSA and the solutions in the niche core. If it is outside the niche radius, put the new solution set in the niche core, otherwise give it up;

Step 4: Continue the next cycle until all local and global optimal solutions are found or the algorithm termination conditions are satisfied.

(2) To solve the problem of difficulty in determining the niche radius, an adaptive niche radius mechanism is proposed. The steps to calculate the niche radius of the multi-peaks function *f* (*x*_1_ *x*_2_ *x*_3_…*x*_d_) are as follows:

Step 1: When there is a solution *X* (*x*_1_ *x*_2_ *x*_3_…*x*_d_) in the niche core, look for the niche radius in the increasing direction of the *i*-th dimension. Increase Δ*R*_i_ along the *x*_i_ direction, and calculate *f* (*x*_1_ *x*_2_ *x*_i_ + Δ*R*_i_…*x*_d_), Δ*R*_i_ is set according to the definition domain of each dimension parameter; Δ*R*_i_ is recommended as shown in Equation (11):

(11) $$\Delta R_{i}=(\frac{1}{20}-\frac{1}{10})\times \|x_{i}^{lb}-x_{i}^{up}\|$$

where $x_{i}^{up}$ and $x_{i}^{lb}$ are the upper and lower limits of definition field of the *i*-th dimension parameter.

Step 2: Determine whether *f* (*x*_1_ *x*_2_ *x*_i_…*x*_d_) > *f* (*x*_1_ *x*_2_ *x*_i_ + Δ*R*_i_…*x*_d_) is satisfied, if so, increase the *i*-th dimension parameter by Δ*R*_i_ again until *f* (*x*_1_ *x*_2_ *x*_i_ + (*n* − 1)Δ*R*_i_…*x*_d_) < *f*(*x*_1_ *x*_2_ *x*_i_ + *n*Δ*R*_i_…*x*_d_). The niche radius in the increasing direction of the *i*-th dimension parameter is *n*Δ*R*_i_;

Step 3: Look for the niche radius in the decreasing direction of the *i*-th dimension. Decrease Δ*R*_i_ along the *x*_i_ direction and calculate *f* (*x*_1_ *x*_2_ *x*_i_ − Δ*R*_i_…*x*_d_). Determine whether *f* (*x*_1_ *x*_2_ *x*_i_…*x*_d_) > *f* (*x*_1_ *x*_2_ *x*_i_ − Δ*R*_i_…*x*_d_) is satisfied, if so, decrease the *i*-th dimension parameter by Δ*R*_i_ again until *f* (*x*_1_ *x*_2_ *x*_i_ + (*m* − 1)Δ*R*_i_…*x*_d_) < *f*(*x*_1_ *x*_2_ *x*_i_ − *m*Δ*R*_i_…*x*_d_).The niche radius in the decreasing direction of the *i*-th dimension is *m*Δ*R*_i_;

Step 4: Available from step 1 to step 3, the niche radius of the *i*-th dimension is *R*_i_ = min(*n*Δ*R*_i_, *m*Δ*R*_i_);

Step 5: Determine the niche radius of other dimensions, then the radius of each dimension of *X* (*x*_1_ *x*_2_ *x*_3_…*x*_d_) is recorded as *R* = (*R*_1_ *R*_2_ *R*_3_…*R*_d_);

(3) In order to improve the accuracy of AFSA optimization, the optimal solution obtained from each execution of AFSA is combined with simulated annealing algorithm to carry out local refinement and optimization. The specific steps are as follows:

Step 1: Set the initial temperature *T*_0_ and the end temperature *Tend* of the simulated annealing algorithm to determine the number of iterations *L* at each temperature;

Step 2: Execute the steps of AFSA to find the best solution *x*_best_;

Step 3: Let *x*_best_ have a random disturbance and get a new solution *x*_best_’;

Step 4: Judge whether *f* (*x*_best_’) is better than *f* (*x*_best_), if so, accept it; otherwise, judge whether exp(−d*f*/*T*) > rand is satisfied, if yes, still accept *x*_best_’, otherwise give up it. Repeat step 3 and step 4 until the termination temperature condition is met where d*f* = *f*(*x*_best_’) − *f*(*x*_best_); rand means a random number between 0 and 1.

### 3.3. Numerical Simulation Analysis

Three classic multi-peak functions are used to test IAFSA. The function expression is shown in Equations (12)–(14).

(12) $$f_{1}(x,y)=2+\sin(19\pi x)+\sin(19\pi y)+x/1.7+y/1.7\;x,\;y\in \left[0,1\right]$$

Features: *f*_1_(*x*, *y*) contains 100 peaks, and the peaks gradually increase from the coordinates (0,0) to (1,1). It is used to test the performance of IAFSA in finding multiple peaks of multi-peak function.

(13) $$f_{2}(x,y)=x\times \sin(4\pi x)-y\times \sin(4\pi y+\pi )+1\;x,\;y\in \left[-1,2\right]$$

Features: *f*_2_(*x*, *y*) contains 36 peaks with unequal spacing. The performance of adaptive niche radius mechanism of IAFSA is tested by this function.

(14) $$f_{3}(x,y)=200-{\left(x^{2}+y-11\right)}^{2}-{\left(y^{2}+x-7\right)}^{2}\;x,\;y\in \left[-6,6\right]$$

Features: *f*_3_(*x*, *y*) contains four global optimal solutions (GOS) with theoretical values of 200. The accuracy of IAFSA is tested by this function.

Before testing, the basic parameters of IAFSA need to be set, the basic parameters are set according to the following principles:

(1) Fish population size. The size of fish population ranges from 50 to 200, if the value is too large, it will increase the time consumption; if the value is too small, the optimization accuracy will be low;
(2) Number of iterations. Similar to the fish population size, the recommended range is 20 to 100;
(3) Visual and step. These two parameters are not sensitive to the optimization effect. Their values are suggested in Equations (15)–(16).
  (15) $$Visual=(\frac{1}{20}-\frac{1}{2})\times \|x_{i}^{lb}-x_{i}^{up}\|$$
  (16) $$S\mathrm{tep}=(\frac{1}{100}-\frac{1}{20})\times \|x_{i}^{lb}-x_{i}^{up}\|$$
(4) Crowding factor. This parameter can range from 0.1 to 0.3;
(5) The recommended value of Δ*R* is shown in Equation (11);
(6) Number of AFSA executions. This value indicates the number of times AFSA is executed when the IAFSA is executed once; the value is greater than the peak numbers of the test function.

According to the above principles, the basic parameter settings of IAFSA are shown in Table 1.

Perform IAFSA 50 times to find the peaks of *f*_1_. In order to compare the performance of SSGA proposed previously [17] and IAFSA, SSGA is also executed 50 times. Limited to space, extract the first five search results. Meanwhile, five groups of AFSA will be executed, and each group will execute AFSA 120 times (ensure that the number of AFSA executions in each group is the same as the number of IAFSA executions each time). The results are shown in Table 2.

**Figure 4:**
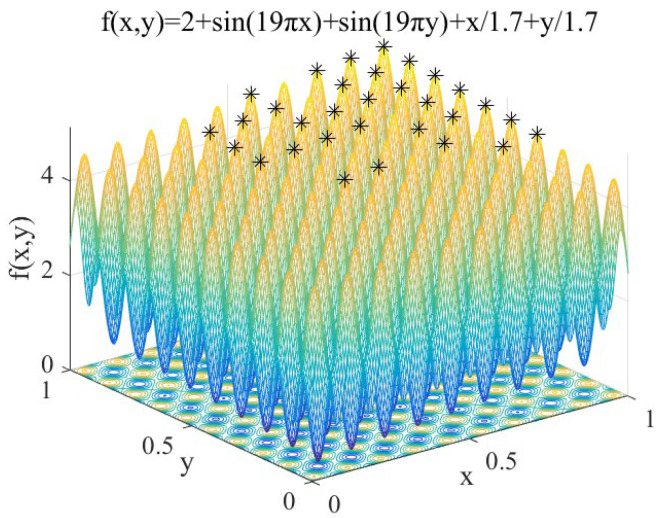
When IAFSA executes AFSA to 30 times.

**Figure 5:**
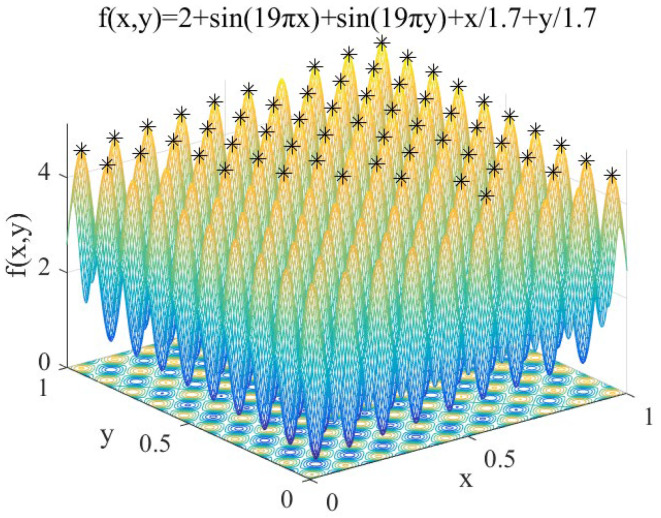
When IAFSA executes AFSA to 60 times.

**Figure 6:**
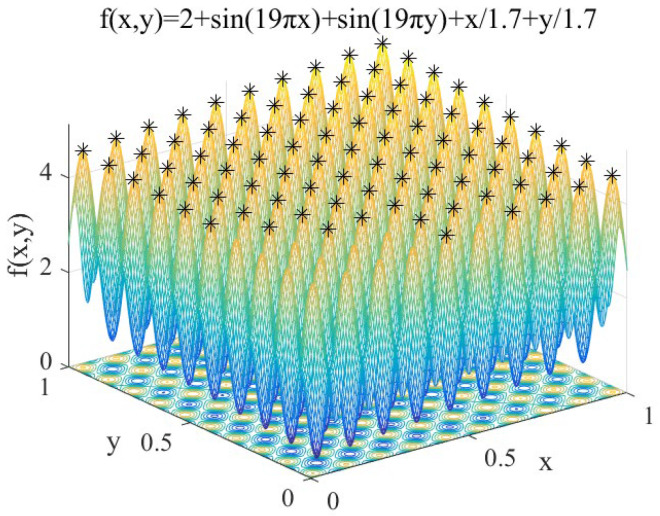
When IAFSA executes AFSA to 90 times.

**Figure 7:**
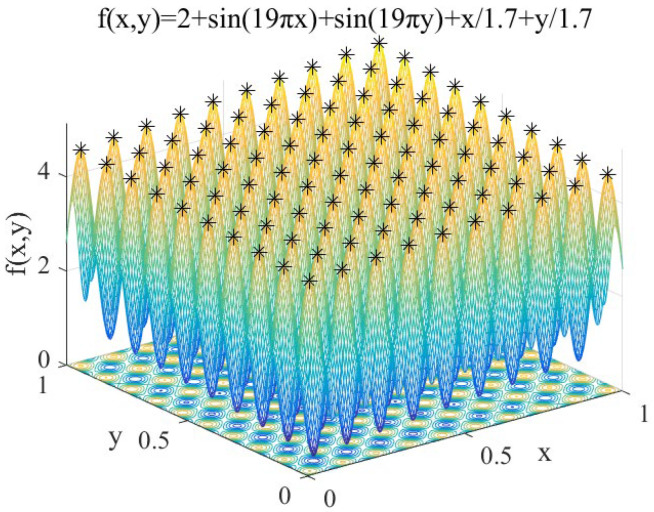
When IAFSA executes AFSA to 120 times.

Record the peak seeking process performed by IAFSA once, and when IAFSA performs AFSA 30 times, 60 times, 90 times, and 120 times. The results are shown in Figure 4, Figure 5, Figure 6 and Figure 7.

From Table 2 and Figure 4, Figure 5, Figure 6 and Figure 7, we can see that the first five times of IAFSA’s execution successfully found one global optimal solution and 99 local optimal solutions of *f*_1_. In fact, the author counted the results of running IAFSA 50 times and found 100 peaks of *f*_1_ successfully each time. However, SSGA can only find one global optimal solution and a few local optimal solutions of *f*_1_, and the number of solutions found is not stable. Meanwhile, the result of running AFSA for each group shows that AFSA can find only one global optimal solution of *f*_1_, and 99 local optimal solutions are missed.

**Figure 8:**
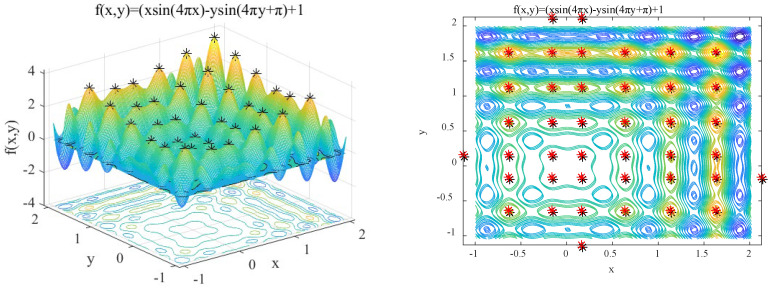
Results of performing IAFSA once to find the peaks of *f*_2_.

In order to test the adaptive niche radius mechanism of IAFSA, IAFSA and fixed niche radius *R* = 0.1, *R* = 0.3, and *R* = 0.5 were executed 50 times to find *f*_2_ peak respectively, and the results of the first 10 times are shown in Table 3. Perform IAFSA once and find the peak of *f*_2_ as shown in Figure 8.

It can be seen from Table 3 that IAFSA using adaptive niche radius mechanism successfully finds one global optimal solution and 35 local optimal solutions of function *f*_2_. In fact, running IAFSA 50 times successfully found 36 peaks of function *f*_2_ each time. When the niche radius is fixed, the niche artificial fish swarm algorithm can find the global optimal solution of function *f*_2_, but it can only find a small number of local optimal solutions, and the number of solutions is not stable.

**Figure 9:**
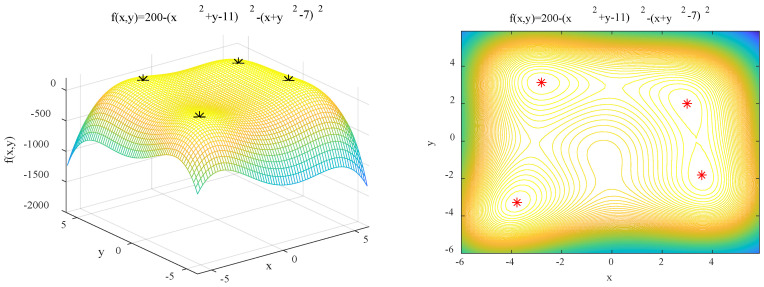
Results of performing IAFSA once to find the peaks of *f*_3._

In order to test the optimization accuracy of IAFSA, IAFSA was run five times, AFSA was run in five groups, and each group was run five times. The statistical results are shown in Table 4, one of the results of running IAFSA is shown in Figure 9.

It can be seen from Table 3 and Figure 9 that IAFSA runs five times and finds four sets of global optimal solutions for *f*_3_ each time. The results of a group of AFSA operations are as follows: *f*_3_ (−3.768343, −3.277694) = 199.993398; *f*_3_ (3.596599, −1.870760) = 199.986524; *f*_3_ (−2.799671, 3.138028) = 199.997172; *f*_3_ (−3.778259, −3.288557) = 199.998502; *f*_3_ (−2.808160, 3.131370) = 199.999699. The results show that running AFSA for many times cannot guarantee the convergence to all the global optimal solutions of the function, and there is the problem of low accuracy in the optimization.

## 4. Case Study of Multi-Alternatives Problem in FEMU

### 4.1. Introduction of ASCE Benchmark Model

**Figure 10:**
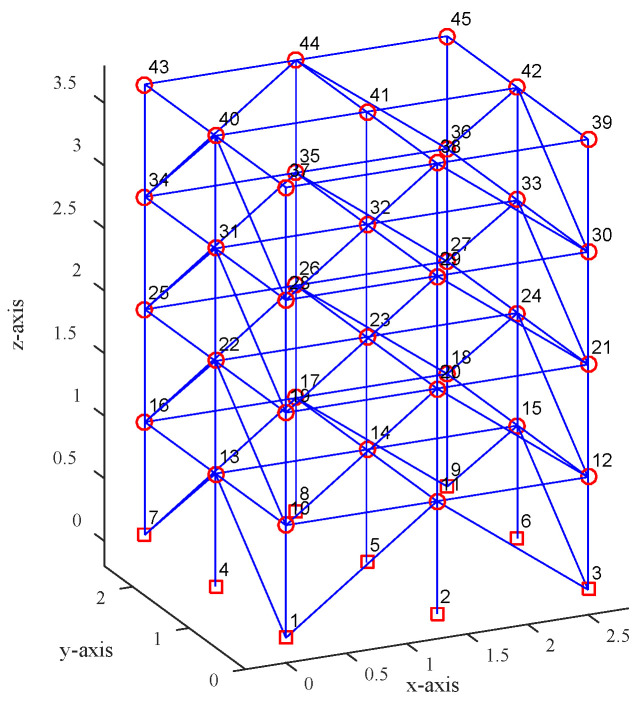
The ASCE benchmark finite element model.

ASCE benchmark model was proposed by Black and Ventura and built in 1998. The purpose of model construction is to be used for structural health monitoring and damage identification, providing a unified test platform for different damage identification methods. The model is a 4-story × 2-span × 2-span frame structure composed of beams, columns, braces, and other components. The size and material characteristics of each component are detailed elsewhere [17], and the model is shown in Figure 10.

**Figure 11:**
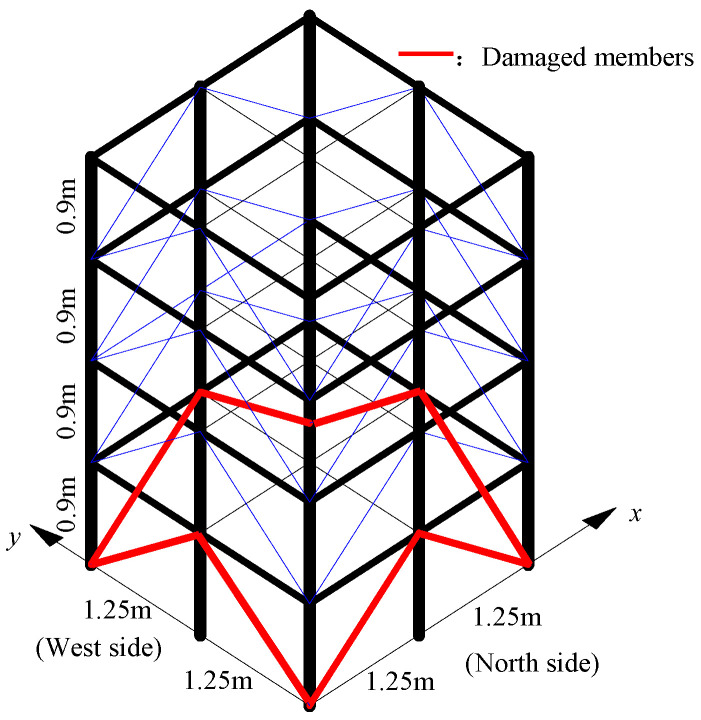
Custom damage of members.

ASCE benchmark model can select 60 degrees of freedom or 120 degrees of freedom mode by modifying the MATLAB code, and also can customize the damage of each member. In this paper, 120 degrees of freedom models are selected. We set the damage to the Young’s modulus of the first layer diagonal brace of the model, and the damaged part of the member is shown in Figure 11. The Young’s modulus values of the damaged members are *E*_1_ = 0.75*E*_0_ (north side), *E*_2_ = 0.5*E*_0_ (east side), *E*_3_ = 0.45*E*_0_ (south side), *E*_4_ = 0.95*E*_0_ (west side). Calculate the first six frequencies of the model before and after the damage, as shown in Table 5.

It can be seen from Table 5 that the maximum relative error of the first six frequencies of the initial model and the damaged model reach 7.12%. The modal assurance criterion (MAC) values are all above 0.947, which show that the first six frequencies of the damage model and the initial model are in the same order mode with high confidence.

### 4.2. Establishment of Kriging Model

**Figure 12:**
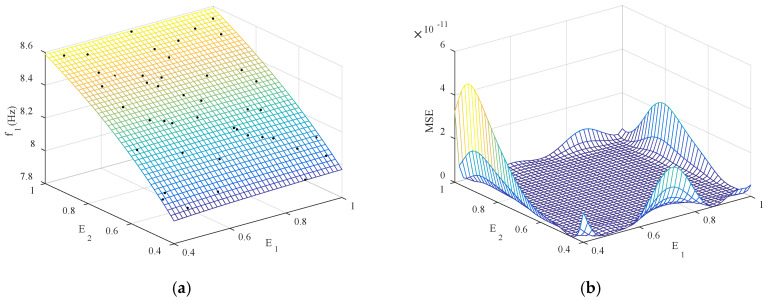
The first frequency in the function of *E*_1_ and *E*_4_: (**a**) Kriging model and data samples; (**b**) the corresponding mean square error (MSE).

Next, 44 sets of sample data were generated using the Latin hypercube design method, and Kriging models with *E*_1_, *E*_2_, *E*_3_, *E*_4_ as input parameters and *f*_1_–*f*_6_ as output were established. To ensure the physical significance of the four updating parameters, when generating sample data, set the parameter range to [40% *E*_0_, *E*_0_], where *E*_0_ represents the initial Young’s modulus of the member. To facilitate data processing, input parameters are normalized. The Kriging model is established based on the sampled data. Figure 12a shows the first frequency in the function of *E*_1_ and *E*_2_, and Figure 12b further shows the MSE value of the Kriging model.

It can be seen from Figure 12 that almost all the sample points fall on the response surface, and the magnitude of the mean square error of the parameters *E*_1_ and *E*_2_ corresponding to *f*_1_ is 10^−11^, which indicates that the Kriging model constructed has high precision. In order to further test the fitting accuracy of *f*_1_–*f*_6_, 50 groups of sample data are regenerated for Kriging model prediction, and the accuracy is tested by using the determination coefficient (R^2^) and root mean square error (RMSE). The test results are shown in Table 6.

It can be seen from Table 6 that R^2^ values are above 0.9988, and RMSE values are all less than 3.99 × 10^−5^, which further indicates that the constructed Kriging model has a high fitting accuracy.

### 4.3. Model Updating and Results Analysis

The updating parameters are *E*_1_–*E*_4_, and the error objective function of fitting value of Kriging model and theoretical value of the damage model is shown in Equation (17).

(17) $$\min f(x)=\min{\sum_{i=1}^{6}(\frac{f_{Kri}^{i}(x)-f_{t}^{i}}{f_{t}^{i}})}^{2},\;x\in [0.4E_{0},E_{0}]$$

where $f_{Kri}^{i}(x)$ represents the *i*-th frequency predicted by Kriging model, $f_{t}^{i}(x)$ represents the *i*-th frequency of damage model, and *x* means the updating parameters.

Running IAFSA, one group of global optimal solutions and three groups of local optimal solutions of the objective function are obtained. The ratio of each parameter after updating to before updating is shown in Table 7.

Four sets of solutions are substituted into ASCE benchmark model to calculate *f*_1_–*f*_6_, and the relative error between the theoretical response corresponding to each set of solutions and the damage model response is calculated as shown in Table 8.

**Figure 13:**
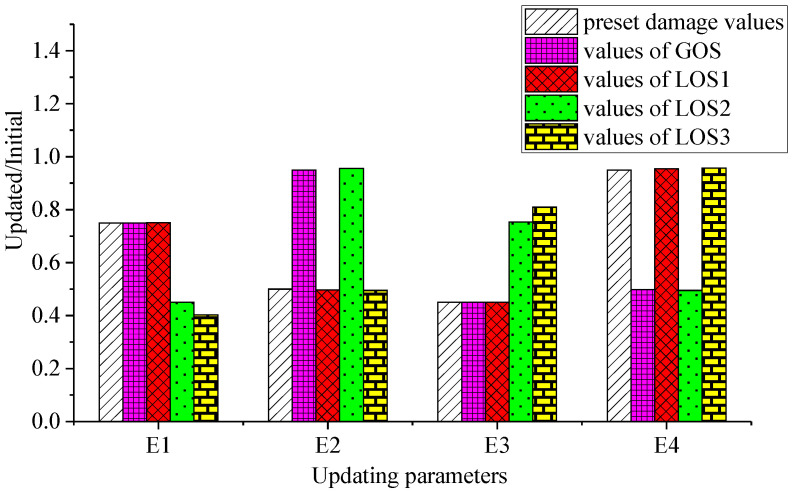
Ratio of parameters corresponding to each group of solutions to initial values.

It is known from Table 8 that the four sets of solutions all effectively reduce the error between the updated model and the damaged model. From the perspective of the degree of error reduction, it is clear that the global optimal solution is the best recommended solution. However, the damage of the ASCE benchmark model is preset by the authors. The values of *E*_1_, *E*_2_, *E*_3_, and *E*_4_ are 0.750 *E*_0_, 0.50 *E*_0_, 0.45 *E*_0_, and 0.95 *E*_0_, respectively. The global optimal solutions obtained by IAFSA are 0.750 *E*_0_, 0.950 *E*_0_, 0.450 *E*_0_, and 0.499 *E*_0_, respectively. Obviously, the global optimal solution is not the damage value preset by the authors. On the contrary, the closest solution to the preset damage value is local optimal solution 1, and the corresponding parameters are 0.751 *E*_0_, 0.497 *E*_0_, 0.451 *E*_0_, and 0.954 *E*_0_. In order to clearly compare the relationship between each group solution and the preset damage value, the ratio of the parameters corresponding to each group of solutions to the initial value is shown in Figure 13. The ordinate in the graph represents the ratio of the corresponding parameters of each group of solutions to the initial value, and the abscissa represents the corresponding parameters of each group of solutions.

Through the case of ASCE benchmark, the authors not only prove that IAFSA can find multiple solutions of objective function in FEMU, but also prove that the global optimal solution in FEMU does not necessarily represent the real parameters of the structure.

## 5. Modal Updating of a Cable-Stayed Footbridge

### 5.1. Engineering Background

**Figure 14:**
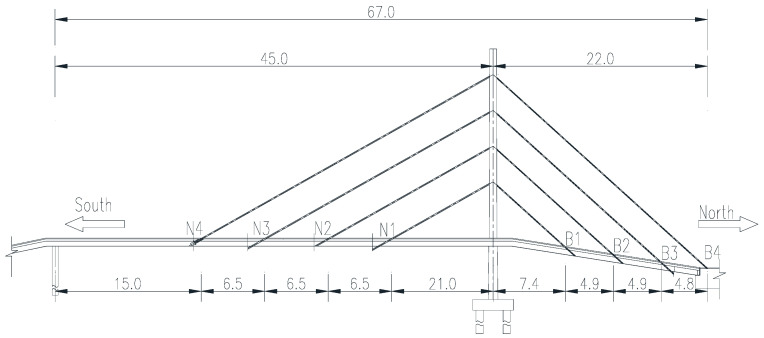
The elevation of pedestrian overpass (unit: m).

The pedestrian bridge in this case is a steel box girder cable-stayed bridge with a total length of 128.8 m, of which the main span is 45 m and the side span is 21 m. The main tower adopts a rectangular steel box girder with a variable cross-section and a height of 24.5 m. The main tower is provided with four parallel cables on both sides along the longitudinal bridge direction, the cable diameter is 115 mm, the design tension of the cables is: *f*_N4_, *f*_B1_ − *f*_B4_ = 430 kN, *f*_N1_ − *f*_N3_ = 355 kN. The elevation of the pedestrian overpass is shown in Figure 14.

**Figure 15:**
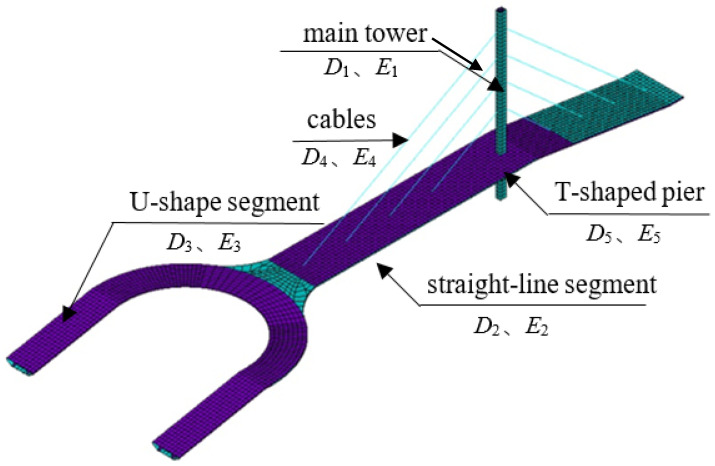
The finite element model (FEM) of the footbridge.

We used Ansys15.0 software to establish the FEM of this bridge, as shown in Figure 15.

**Figure 16:**
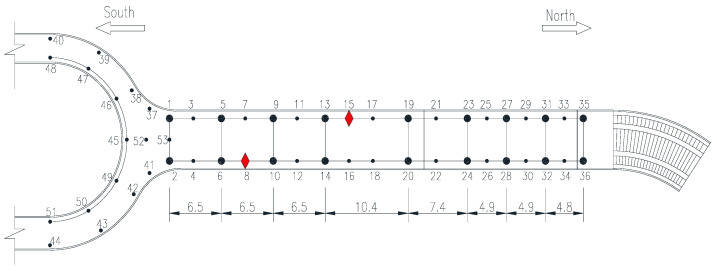
Layout of dynamic test points of footbridge (unit: m).

In order to obtain the dynamic response, the environmental vibration of the bridge was tested. A total of 53 measuring points are set in the whole bridge, which are divided into 17 measurement batches. In order to ensure the comparability and consistency of the measured data of each batch, each batch includes two reference points, namely point 8 and point 15. The data acquisition time of each batch is 15 min, and the acquisition frequency is set to 200 Hz. The layout of dynamic test points of the footbridge is shown in Figure 16.

**Figure 17:**
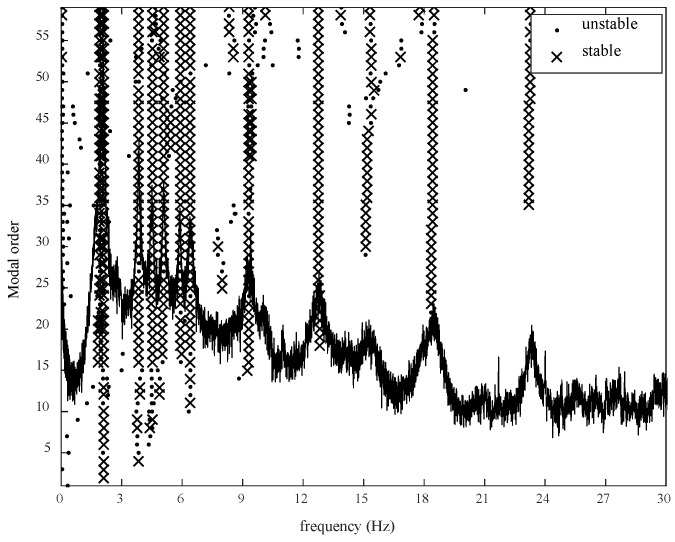
Stability graph of stochastic subspace identification.

The modal information of the bridge was identified by the stochastic subspace method, and the order was determined by the stability diagram. Take the identified frequency as shown in Figure 17, at the same time, extract the corresponding modal response under the initial modeling parameters of the finite element model as shown in Table 9.

### 5.2. Updating Parameters Selection and Establishment of Kriging Model

When selecting the updating parameters, the following aspects are mainly considered:

(1) The steel box girder is a prefabricated component, so it is difficult to change the section area or the moment of inertia due to the size deviation of the section;
(2) The tension of cable may relax under frequent loads;
(3) There are different degrees of corrosion in straight-line segment and U-shape segment of steel box girder, which greatly affects the Young’s modulus and density of beam;
(4) The actual stiffness of the main tower should be greater than the design value when the section thickness of the main tower at the connection of the cable and the main tower increases;
(5) T-shaped pier as a member supporting the main beam; its Young’s modulus and density may change with the increase of service life.

**Figure 18:**
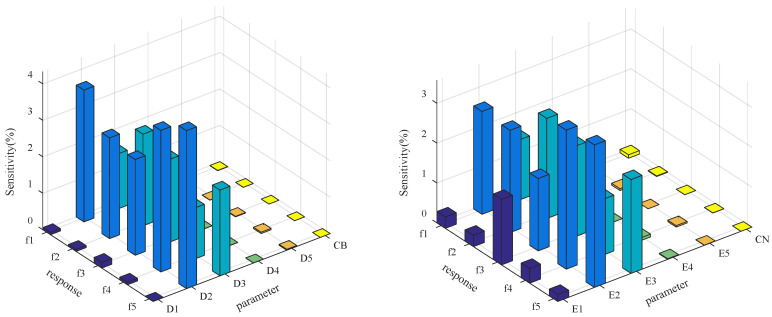
Histogram of parameter sensitivity analysis.

Therefore, the following parameters are preliminarily selected as the updating parameters: density and Young’s modulus of main tower (denoted by *D*_1_ and *E*_1_), density and Young’s modulus of steel box girder in straight-line segment (denoted by *D*_2_ and *E*_2_), density and Young’s modulus of steel box girder in U-shape segment (denoted by *D*_3_ and *E*_3_), density and Young’s modulus of the cables (denoted by *D*_4_ and *E*_4_), density and Young’s modulus of T-shaped pier (denoted by *D*_5_ and *E*_5_), and cable tension (denoted by *CN* and *CB*). For the preselected parameters, set 10% change based on the initial values, and substitute them into the finite element model to calculate the frequency change percentage. The sensitivity of each parameter to the extracted modal frequency is shown in Figure 18.

**Figure 19:**
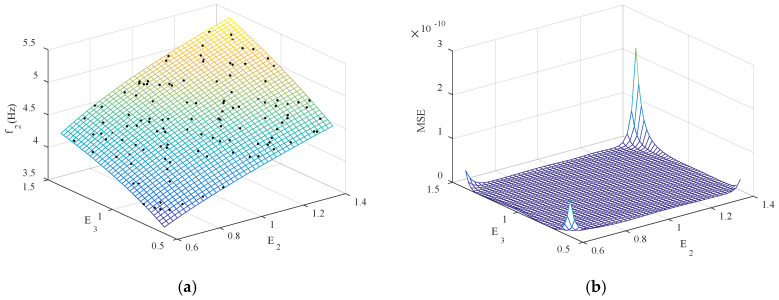
The frequency of *f*_2_ in the function of *E*_2_ and *E*_3_: (**a**) Kriging model and data samples; (**b**) the corresponding MSE.

**Figure 20:**
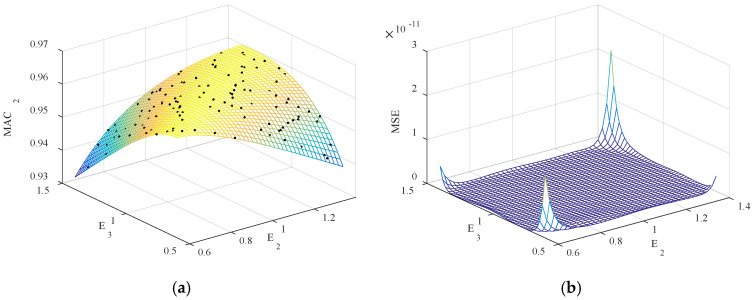
The value of MAC_2_ in the function of *E*_2_ and *E*_3_: (**a**) Kriging model and data samples; (**b**) the corresponding MSE.

From Figure 18, *E*_1_, *E*_2_, *E*_3_, *D*_2_, *D*_3_ with high sensitivity to the bridge’s frequencies are selected as the updating parameters. The initial values of the five updating parameters are set as 2.06 × 10^5^ MPa, 2.06 × 10^5^ MPa, 2.06 × 10^5^ MPa, 7850 kg/m^3^, and 7850 kg/m^3^, respectively. Use LHD to generate 80 groups of updating parameter samples. To ensure that the samples of the parameters have physical significance, set the range of each parameter interval as [0.6 *X*_0_, 1.4 *X*_0_]. The samples are substituted into the FEM to extract the frequencies, and then the Kriging model, indicating the relationship between the updating parameters and modal frequencies/MAC values, is established based on a regression analysis of the data samples. Figure 19 and Figure 20 demonstrate the typical result of *f*_2_ and MAC_2_ in the function of *E*_2_ and *E*_3_, along with the corresponding MSE. The dots in Figure 19a are the data samples generated by LHD, while the Kriging model generates the surface. The MSE values are smaller than 3 × 10^−10^, indicating that the surrogate model is quite accurate.

### 5.3. Model Updating and Result Analysis

The error objective function of fitting value of Kriging model and measured response is shown in Equation (18).

(18) $$\min f(x)=\min(\alpha_{1}{\sum_{i=1}^{4}(\frac{f_{Kri}^{i}(x)-f_{t}^{i}}{f_{t}^{i}})}^{2}+\alpha_{2}{\sum_{i=1}^{4}(\frac{1-\sqrt{MAC_{i}(x)}}{MAC_{i}(x)})}^{2})$$

where *x* means the updating parameters, and α_1_ and α_2_ are the weight coefficients; in this case, α_1_ = 1, α_2_ = 1.$f_{Kri}^{i}(x)$ represents the frequency calculated by Kriging model,$f_{t}^{i}(x)$ represents the measured values of frequencies. $MAC_{i}(x)$ means the confidence degree that the theoretical mode and the measured mode are of the same order. It should be noted that the measured value of *f*_5_ is not a constraint condition of the objective function but is used to verify the results of the finite element model updating.

Running IAFSA, one group of global optimal solutions and fourteen groups of local optimal solutions of the objective function are obtained. Limited to space, a representative group of global optimal solutions and three groups of local optimal solutions are selected, as shown in Table 10. Table 11 show the difference of parameters before and after updating.

The corresponding parameters of each group of solutions are respectively substituted into the finite element model to extract the 1st, 2nd, 3rd, and 6th vertical bending modes and 1st torsional mode, and the relative error between the theoretical response corresponding to each group of solutions and the measured value is calculated as shown in Table 12. The MAC values under each group of solutions are shown in Table 13.

From Table 12, it can be seen that the four groups of solutions can effectively reduce the error between the theoretical value and the measured value of the frequencies, and the global optimal solution has the most significant reduction effect. However, it is not comprehensive that the only criterion for parameters selection is to reduce the error between the theoretical values and the measured values of the model. Table 11 shows the change degree of parameters corresponding to each group of solutions before and after update. The parameters of the global optimal solution are as follows: the density of the straight-line segment of beam is reduced by 36.94%, the Young’s modulus is reduced by 39.81%, while the density and Young’s modulus of U-shaped segment are increased to 27.26% and 39.81%, respectively. Considering the actual situation of bridge diseases and combined with engineering experience, the corrosion of straight-line segment of the main beam is more serious than that of U-shaped segment. However, from the practical engineering point of view, the reduction of the Young’s modulus and density of the main beam to nearly 40% is a small probability event. The reasons are as follows:

(1) There is no obvious defect in the section of steel box girder, and the density and inertia moment of the girder will not be greatly reduced;
(2) There are auxiliary facilities such as pavement and steel guardrail on the bridge deck system, which will buffer the decrease of the density and stiffness of the main beam caused by the corrosion of the steel structure to some extent.

**Figure 21:**
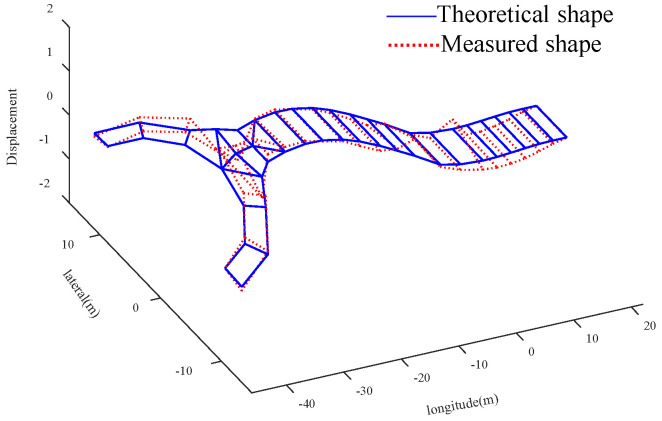
Comparison of the shape of 1st ↕

**Figure 22:**
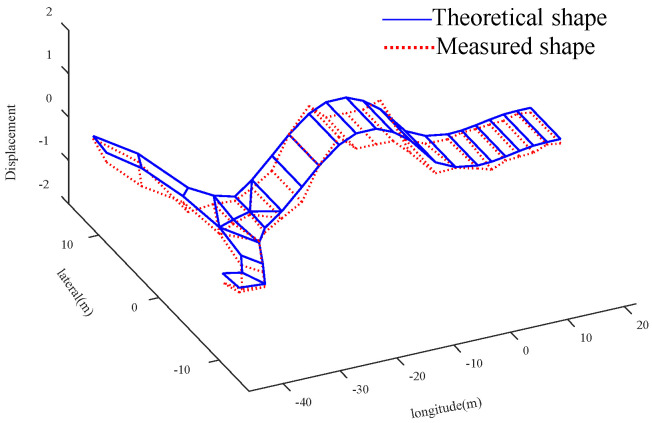
Comparison of the shape of 2nd ↕

**Figure 23:**
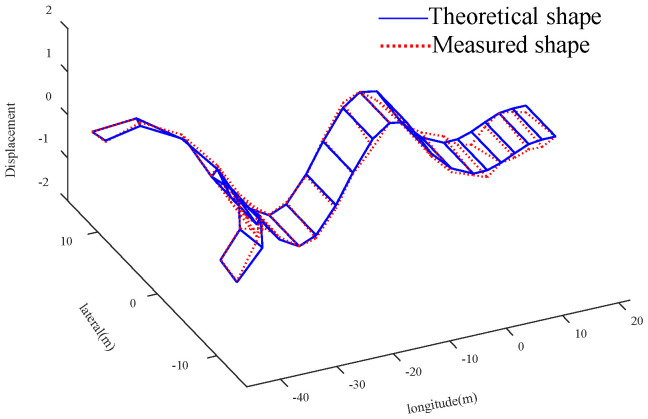
Comparison of the shape of 3rd ↕

**Figure 24:**
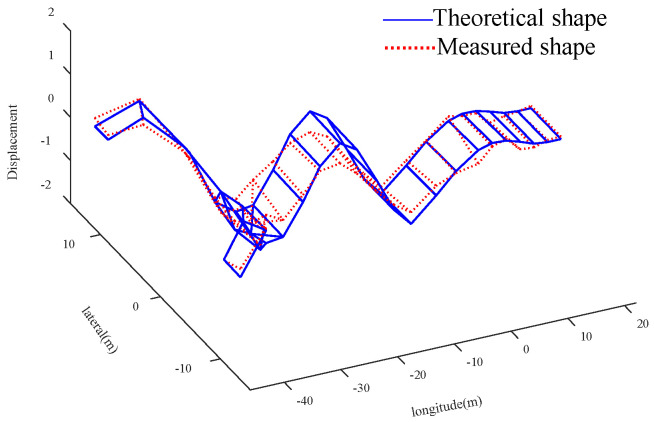
Comparison of the shape of 6th ↕.

Compared with the global optimal solution, the parameter value corresponding to the sub optimal solution 2 is more consistent with the actual bridge condition. The analysis is as follows: Compared with the initial value, the elastic modulus of the main tower increases by 28.16%, the density and elastic modulus of the straight-line segment of beam decrease by 12.87% and 7.77%, and the density and elastic modulus of U-shaped segment increase by 12.48% and 17.48%, respectively. Due to the increase of the section thickness of the connection between the cable and the main tower, and the existence of lifting lugs, stiffeners and other components, it is possible that the elastic modulus of the main tower is larger than the initial value. It should be noted that the measured value of *f*_5_ (the 6-th vertical bending mode) is used to verify the results of the finite element model updating. Four groups of solutions are substituted into the FEM to calculate *f*_5_ and MAC_5_. For the global optimal solution, the error between the theoretical value and the measured value of the 6-th vertical bending frequency is reduced from 3.41% to 2.88%, and the MAC_5_ value is 0.950, for suboptimal solution 2, the error is reduced to 0.07%, and the corresponding MAC_5_ value is 0.955. The graphs of the theoretical and experimental mode shapes of the vertical bending modes corresponding to the suboptimal solution 2 are shown in Figure 21, Figure 22, Figure 23 and Figure 24. Therefore, in this case, the suboptimal solution 2 is selected as the solution that best represents the bridge parameters.

## 6. Conclusions

(1) An improved artificial fish swarm algorithm is proposed, which introduces niche technology, adaptive niche radius mechanism, and uses simulated annealing algorithm to locally refine and optimize in the later stage of the algorithm. Through three typical functions, IAFSA is verified to have high efficiency, high precision, and intelligent search for multiple solutions of multiple peak functions.
(2) In the case of ASCE benchmark finite element model updating, one set of global optimal solutions and three sets of local optimal solutions of the objective function are obtained. By comparing the preset damage value with the four sets of solutions, it is found that the parameters corresponding to the global optimal solution are different from the preset values, while the suboptimal solution 1 can match well with the preset values, which proves that the global optimal solution does not necessarily represent the actual parameters value of the structure.
(3) IAFSA is applied to the model updating of a pedestrian bridge, and 15 groups of solutions are obtained to reduce the error objective function. Combined with the actual bridge situation and reanalysis technology, the suboptimal solution 2 is selected as the most representative solution of the real parameters of the structure, other solutions are used as alternatives for reliability analysis and risk assessment of the bridge.
(4) In two cases of model updating, Kriging model is used instead of finite element model, which improves the calculation efficiency of FEMU on the premise of meeting the accuracy of fitting frequencies and MAC values.

## Figures and Tables

**Table 1:** Parameters setting of improved artificial fish swarm algorithm (IAFSA) corresponding to the test functions.

Parameter Setting	*f*_1_	*f*_2_	*f*_3_
Fish population size	50	50	50
Number of iterations	20	20	30
Visual	0.3	0.75	0.75
Step	0.015	0.135	0.15
Crowding factor	0.1	0.1	0.1
Δ*R*	0.05	0.15	0.6
*T*_0_	1500	1500	1500
Tend	0.01	0.01	0.01
Chain length	200	200	200
Number of AFSA executions	120	45	5

**Table 2:** The results of optimizing *f*_1._

Number of Runs	IAFSA		SSGA		Number of Groups Running	AFSA	
	Number of GOS Found	Number of LOS Found	Number of GOS Found	Number of LOS Found		Number of GOS Found	Number of LOS Found
First time	1	99	1	5	1st group	1	0
Second time	1	99	1	6	2nd group	1	0
Third time	1	99	1	4	3rd group	1	0
Fourth time	1	99	1	5	4th group	1	0
Fifth time	1	99	1	5	5th group	1	0

GOS: global optimal solutions, LOS: local optimal solutions.

**Table 3:** Results of optimizing *f*_2_ with different niche radius.

Number of Runs	Adaptive Radius		*R* = 0.1		*R* = 0.3		*R* = 0.5	
	GOS Found	LOS Found	GOS Found	LOS Found	GOS Found	LOS Found	GOS Found	LOS Found
1	1	35	1	6	1	31	1	23
2	1	35	1	6	1	31	1	21
3	1	35	1	10	1	30	1	21
4	1	35	1	9	1	31	1	23
5	1	35	1	12	1	29	1	22
6	1	35	1	11	1	31	1	24
7	1	35	1	10	1	31	1	21
8	1	35	1	9	1	31	1	21
9	1	35	1	12	1	29	1	23
10	1	35	1	7	1	31	1	24

**Table 4:** Results of IAFSA and AFSA optimize *f*_3._

Number of Runs	Peaks Found	Average of Peak Coordinates	Average of Fitness
First time	4	(3.000478, 1.999398)	199.999991
Second time	4	(3.584221, −1.848550)	199.999995
Third time	4	(−2.804652, 3.131278)	199.999993
Fourth time	4	(−3.779525, −3.283265)	199.999998
Fifth time	4		

**Table 5:** First 6 frequencies of initial model and damaged model. MAC: modal assurance criterion.

Mode	Initial FEM *f*_a_ (Hz)	Damaged FEM *f*_t_ (Hz)	Relative Difference (%)	MAC
*f*_1_	8.588	8.051	6.25	0.982
*f*_2_	9.181	8.638	5.91	0.989
*f*_3_	14.580	13.542	7.12	0.972
*f*_4_	23.452	22.404	4.47	0.989
*f*_5_	25.946	24.767	4.54	0.994
*f*_6_	36.813	36.222	1.61	0.947

**Table 6:** Determination coefficient (R^2^) and root mean square error (RMSE) values corresponding to the first six frequencies.

Inspecting Method	*f*_Kri_^1^	*f*_Kri_^2^	*f*_Kri_^3^	*f*_Kri_^4^	*f*_Kri_^5^	*f*_Kri_^5^
R^2^	1.0000	0.9998	0.9999	1.0000	1.0000	0.9988
RMSE	1.95 × 10^−0.5^	3.99 × 10^−0.5^	2.48 × 10^−0.5^	2.45 × 10^−0.6^	5.86 × 10^−0.6^	2.87 × 10^−0.6^

**Table 7:** Comparison of parameters before and after updating.

Updating Parameters	*E*_0_	GOS		LOS1		LOS2		LOS3	
		*E’*	*E’*/*E*_0_	*E’*	*E’*/*E*_0_	*E’*	*E’*/*E*_0_	*E’*	*E’*/*E*_0_
*E*_1_/(× 10^5^ MPa)	2.000	1.500	0.750	1.502	0.751	0.900	0.450	0.804	0.402
*E*_2_/(× 10^5^ MPa)	2.000	1.900	0.950	0.994	0.497	1.912	0.956	0.990	0.495
*E*_3_/(× 10^5^ MPa)	2.000	0.900	0.450	0.902	0.451	1.506	0.753	1.620	0.810
*E*_4_/(× 10^5^ MPa)	2.000	0.998	0.499	1.908	0.954	0.990	0.495	1.914	0.957

Note: *E*_0_ stands for initial value, *E’* stands for updated value.

**Table 8:** The error of frequency between updated model and damaged model.

Mode	Initial Model Hz	Damaged Model		Updated by GOS		Updated by LOS1		Updated by LOS2		Updated by LOS3	
		Hz	Error %	Hz	Error %	Hz	Error %	Hz	Error %	Hz	Error %
*f*_1_	8.051	8.588	6.25	8.050	0.01	8.050	0.01	8.049	0.03	8.047	0.05
*f*_2_	8.638	9.181	5.91	8.638	0	8.639	0.01	8.640	0.02	8.630	0.09
*f*_3_	13.542	14.580	7.12	13.541	0	13.543	0.01	13.544	0.02	13.545	0.02
*f*_4_	22.404	23.452	4.47	22.403	0	22.405	0.01	22.406	0.01	22.406	0.01
*f*_5_	24.767	25.946	4.54	24.766	0	24.769	0.01	24.771	0.02	24.772	0.02
*f*_6_	36.222	36.813	1.61	36.221	0	36.220	0.00	36.218	0.01	36.218	0.01

**Table 9:** Comparison between measured frequencies and theoretical frequencies.

Mode	Type	Theoretical Frequency (Hz)	Measured Frequency (Hz)	Relative Difference (%)	*MAC*
*f*_1_	1st ↕	1.92	2.08	7.69	0.954
*f*_2_	2nd ↕	4.58	4.60	0.43	0.959
*f*_3_	1st ↺	6.06	6.45	6.05	0.972
*f*_4_	3rd ↕	9.10	9.33	2.47	0.962
*f*_5_	6th ↕	14.74	15.26	3.41	0.952

Note: ↕ stands for vertical bending modes, ↺ stands for torsion mode, MAC: modal assurance criterion.

**Table 10:** Comparison of parameters before and after updating.

Updating Parameters	Initial Values	GOS	LOS1	LOS2	LOS3
		Updated Values	Updated Values	Updated Values	Updated Values
*E*_1_/(×10^5^ MPa)	2.06	2.54	1.83	2.64	1.80
*D*_2_/(×10^3^ kg/m^3^)	7.85	4.95	6.67	6.84	7.09
*E*_2_/(×10^5^ MPa)	2.06	1.24	1.78	1.90	1.98
*D*_3_/(×10^3^ kg/m^3^)	7.85	9.99	10.17	8.83	10.37
*E*_3_/(×10^3^ MPa)	2.06	2.88	2.88	2.42	2.88

**Table 11:** Difference of parameters before and after updating.

Updating Parameters	Initial Values	GOS	LOS1	LOS2	LOS3
		Difference %	Difference %	Difference %	Difference %
*E*_1_/(×10^5^ MPa)	2.06	23.30	−11.17	28.16	−12.62
*D*_2_/(×10^3^ kg/m^3^)	7.85	−36.94	−15.03	−12.87	−9.68
*E*_2_/(×10^5^ MPa)	2.06	−39.81	−13.59	−7.77	−3.88
*D*_3_/(×10^3^ kg/m^3^)	7.85	27.26	29.55	12.48	32.10
*E*_3_/(×10^5^ MPa)	2.06	39.81	39.81	17.48	39.81

**Table 12:** The error between theoretical frequencies and measured values corresponding to each group of solutions.

Mode	Experimental Hz	Initial FEM		Updated by GOS		Updated by LOS1		Updated by LOS2		Updated by LOS3	
		Hz	Error%	Hz	Error %	Hz	Error %	Hz	Error %	Hz	Error %
*f*_1_	2.08	1.92	7.69	2.03	2.40	2.00	3.85	2.00	3.85	2.00	3.72
*f*_2_	4.6	4.58	0.43	4.65	1.09	4.69	1.96	4.71	2.39	4.71	2.49
*f*_3_	6.45	6.06	6.05	6.45	0.00	6.40	0.78	6.39	0.93	6.37	1.18
*f*_4_	9.33	9.10	2.47	9.34	0.11	9.35	0.19	9.41	0.86	9.44	1.16
*f*_5_	15.26	14.74	3.41	14.82	2.88	15.06	1.30	15.25	0.07	15.25	0.03

**Table 13:** MAC values corresponding to each group of solutions.

Mode Type	Initial FEM	GOS	LOS1	LOS2	LOS3
1st ↕	0.954	0.954	0.954	0.955	0.954
2nd ↕	0.959	0.956	0.950	0.947	0.951
1st ↺	0.972	0.990	0.991	0.988	0.991
3rd ↕	0.962	0.980	0.981	0.980	0.980
6th ↕	0.952	0.950	0.953	0.955	0.952

Note: ↕ stands for vertical bending modes, ↺ stands for torsion mode.
